# Supplementary material for: Simultaneous brain, brainstem, and spinal cord pharmacological-fMRI reveals involvement of an endogenous opioid network in attentional analgesia
Source: eLife. 2022 Jan 26;11:e71877. doi: 10.7554/eLife.71877 (PMC8843089; doi:10.7554/eLife.71877)
Supplement: Figure 2—figure supplement 1—source data 1. [file elife-71877-fig2-figsupp1-data1.docx]

Placebo vs Reboxetine 3 way ANOVA

| ANOVA table | F (DFn, DFd) | P value |
| --- | --- | --- |
| Drug | F (1, 38) = 3.417 | P=0.072 |
| Temperature | F (1, 38) = 207.2 | P<0.0001 |
| Task | F (1, 38) = 7.320 | P=0.010 |
| Drug x Temperature | F (1, 38) = 5.060 | P=0.030 |
| Drug x Task | F (1, 38) = 0.1738 | P=0.68 |
| Temperature x Task | F (1, 38) = 14.86 | P=0.0004 |
| Drug x Temperature x Task | F (1, 38) = 0.3496 | P=0.58 |

Placebo vs Naltrexone 3 way ANOVA

| ANOVA table | F (DFn, DFd) | P value |
| --- | --- | --- |
| Drug | F (1, 38) = 0.002508 | P=0.96 |
| Temperature | F (1, 38) = 196.5 | P<0.0001 |
| Task | F (1, 38) = 2.928 | P=0.095 |
| Drug x Temperature | F (1, 38) = 0.7509 | P=0.39 |
| Drug x Task | F (1, 38) = 1.529 | P=0.22 |
| Temperature x Task | F (1, 38) = 4.583 | P=0.039 |
| Drug x Temperature x Task | F (1, 38) = 1.650 | P=0.20 |
