# Supplementary material for: Simultaneous brain, brainstem, and spinal cord pharmacological-fMRI reveals involvement of an endogenous opioid network in attentional analgesia
Source: eLife. 2022 Jan 26;11:e71877. doi: 10.7554/eLife.71877 (PMC8843089; doi:10.7554/eLife.71877)
Supplement: Figure 3—source data 1. [file elife-71877-fig3-data1.docx]

| Voxels | MAX | X (mm) | Y (mm) | Z (mm) | Atlas labels |
| --- | --- | --- | --- | --- | --- |
| **Main effect of temperature** | | | | | |
| 3295 | 12.4 | 36 | 12 | -10 | 74% Insular Cortex |
| 531 | 6.56 | -32 | -14 | 22 | 12% Frontal Operculum Cortex, |
| 436 | 7.53 | -34 | 14 | 16 | 47% Precentral Gyrus, 26% Central Opercular Cortex |
| 246 | 6.86 | -58 | 0 | 8 | 10% Occipital Fusiform Gyrus |
| 131 | 6.49 | 38 | -66 | -22 | 25% Juxtapositional Lobule Cortex (formerly Supplementary Motor Cortex) |
| 65 | 6.83 | 4 | -38 | -46 | 100% Brain-Stem |
| 61 | 6.24 | -40 | -2 | -2 | 10% Right V |
| 47 | 5.81 | 24 | -20 | 64 | 72% Frontal Pole |
| 43 | 8.07 | -20 | 52 | 26 | 43% Supramarginal Gyrus, anterior division, 20% Parietal Operculum Cortex, 11% Postcentral Gyrus |
| 16 | 6.11 | -4 | -68 | -40 | 46% Frontal Operculum Cortex, 15% Insular Cortex |
| 13 | 5.68 | -34 | 22 | 6 | 31% Insular Cortex |
| 11 | 5.43 | -36 | -20 | -2 | 11% Frontal Operculum Cortex, 5% Inferior Frontal Gyrus, pars triangularis |
| 10 | 5.43 | 36 | 22 | 14 | 29% Middle Frontal Gyrus, 8% Inferior Frontal Gyrus, pars triangularis, 7% Inferior Frontal Gyrus, pars opercularis |
| 10 | 6.94 | 40 | 22 | 26 | 10% Supramarginal Gyrus, posterior division, 6% Superior Parietal Lobule |
| 8 | 5.42 | 24 | -46 | -22 | 45% Frontal Pole, 12% Inferior Frontal Gyrus, pars triangularis, 11% Middle Frontal Gyrus |
| 5 | 5.87 | 44 | 36 | 14 | 43% Frontal Pole, 5% Inferior Frontal Gyrus, pars triangularis |
| 4 | 5.38 | 18 | -12 | 28 | 6% Precuneus Cortex |
| 4 | 5.23 | -18 | -60 | 36 | 11% Postcentral Gyrus |
| 4 | 5.79 | -22 | -62 | -38 | 20% Frontal Orbital Cortex |
| 3 | 5.11 | -32 | 32 | -4 | 45% Frontal Operculum Cortex, 19% Frontal Orbital Cortex, 12% Insular Cortex |
| 3 | 12.6 | 0 | -36 | -6 | 71% Brain-Stem |
| 2 | 5.57 | 20 | 56 | 20 | 5% Temporal Occipital Fusiform Cortex |
| 2 | 5.03 | 46 | -52 | -28 | 56% Frontal Pole |
| 2 | 8.8 | -40 | 42 | 4 | 34% Frontal Pole |
| 2 | 5.93 | 22 | 50 | 20 | 23% Temporal Occipital Fusiform Cortex, 23% Temporal Fusiform Cortex, posterior division |
| 2 | 5.16 | -32 | -44 | -24 | 23% Precuneus Cortex, 15% Postcentral Gyrus, 12% Precentral Gyrus, 10% Cingulate Gyrus, posterior division |
| 2 | 8.66 | 2 | -36 | 52 | 61% Frontal Pole, 9% Middle Frontal Gyrus, 6% Inferior Frontal Gyrus, pars triangularis |
| 2 | 5.74 | 14 | -70 | -42 | 83% Frontal Pole |
| 2 | 5.7 | 44 | 40 | 14 | 48% Lateral Occipital Cortex, inferior division |
| 2 | 5.47 | -28 | 60 | 14 | 24% Lateral Occipital Cortex, inferior division |
| 2 | 5.52 | 52 | -70 | -16 | 52% Cingulate Gyrus, anterior division, 16% Paracingulate Gyrus |
| 2 | 5.03 | -34 | -80 | -2 | 48% Occipital Pole, 8% Lateral Occipital Cortex, inferior division |
| 2 | 5.03 | 24 | -22 | 24 | 32% Parietal Operculum Cortex, 17% Supramarginal Gyrus, anterior division, 6% Planum Temporale |
| 2 | 5.09 | 26 | -94 | -8 | 10% Lingual Gyrus, 5% Occipital Fusiform Gyrus |
| 2 | 5.06 | 10 | -48 | -22 | 10% Frontal Pole, 5% Middle Frontal Gyrus |
| 2 | 5.68 | 8 | -82 | -20 | 35% Supramarginal Gyrus, anterior division |
| 1 | 5.02 | -22 | -4 | 32 | 14% Supramarginal Gyrus, posterior division, 7% Planum Temporale, 6% Angular Gyrus |
| 1 | 5.79 | -32 | 36 | 18 | 13% Cingulate Gyrus, anterior division |
| 1 | 5.15 | 68 | -22 | 30 | 35% Supramarginal Gyrus, anterior division |
| 1 | 5.4 | -48 | -46 | 20 | 14% Supramarginal Gyrus, posterior division, 7% Planum Temporale, 6% Angular Gyrus |
| 1 | 5.26 | 12 | 6 | 34 | 13% Cingulate Gyrus, anterior division |
| 1 | 5.06 | 22 | 48 | 26 | ,56% Frontal Pole |
| 1 | 7.82 | 36 | -32 | 16 | 37% Planum Temporale, 9% Parietal Operculum Cortex |
| 1 | 11.5 | 40 | 22 | 44 | 51% Middle Frontal Gyrus |
| 1 | 8.47 | 28 | 62 | 20 | 66% Frontal Pole |
| 1 | 9.91 | -68 | -38 | 36 | 7% Supramarginal Gyrus, anterior division |
| 1 | 7.81 | -40 | -6 | 36 | 10% Precentral Gyrus |
| 1 | 5.93 | 6 | -26 | 24 | 9% Cingulate Gyrus, posterior division |
| 1 | 5.02 | -30 | 40 | 22 | ,38% Frontal Pole, 12% Middle Frontal Gyrus |
| 1 | 5.17 | 14 | -36 | -30 | 20% Brain-Stem |
| 1 | 6.04 | -14 | -34 | -18 | 13% Parahippocampal Gyrus, posterior division |
| 1 | 5.36 | -2 | -42 | -16 | 10% Brain-Stem |
| 1 | 5.38 | 36 | 12 | -14 | 65% Insular Cortex |
| 1 | 5.13 | -2 | -88 | -12 | 48% Lingual Gyrus, 7% Occipital Pole, 4% Intracalcarine Cortex |
| 1 | 5.69 | 48 | -78 | -12 | 70% Lateral Occipital Cortex, inferior division |
| 1 | 5.19 | -28 | 16 | -12 | 33% Insular Cortex, 20% Frontal Orbital Cortex |
| 1 | 5.04 | 20 | -30 | 64 | 34% Postcentral Gyrus, 31% Precentral Gyrus |
| 1 | 5.2 | -46 | 24 | -12 | 73% Frontal Orbital Cortex |
| 1 | 5.13 | -4 | -76 | -10 | 61% Lingual Gyrus |
| 1 | 5.26 | -34 | -68 | -2 | 7% Lateral Occipital Cortex, inferior division, 5% Occipital Fusiform Gyrus |
| 1 | 5.17 | -28 | -68 | 2 | 5% Intracalcarine Cortex |
| 1 | 5.01 | -34 | -28 | 2 | 3% Insular Cortex |
| 1 | 5.07 | -14 | -88 | 4 | 22% Intracalcarine Cortex, 8% Occipital Pole, 3% Lingual Gyrus |
| 1 | 5.15 | -44 | -18 | 4 | 53% Heschl's Gyrus (includes H1 and H2), 6% Planum Polare |
| 1 | 5.01 | -46 | -8 | 6 | 59% Central Opercular Cortex, 8% Heschl's Gyrus (includes H1 and H2), 7% Planum Polare |
| 1 | 5.5 | -36 | 50 | 6 | 64% Frontal Pole |
| 1 | 5.38 | -34 | -76 | 8 | 12% Lateral Occipital Cortex, inferior division |
| 1 | 5.25 | -54 | -40 | 14 | 26% Planum Temporale, 15% Supramarginal Gyrus, posterior division, 11% Superior Temporal Gyrus, posterior division |
| 1 | 5.09 | -34 | 18 | -12 | 48% Frontal Orbital Cortex, 28% Insular Cortex |
| **Negative main effect of temperature** | | | | | |
| 25 | 5.25 | -4 | 46 | -14 | 81% Frontal Medial Cortex, 11% Paracingulate Gyrus |
| 1 | 6.15 | 8 | 30 | -12 | 27% Subcallosal Cortex, 12% Frontal Medial Cortex |
| **Main effect of task** | | | | | |
| 2585 | 7.81 | 46 | -46 | -8 | 17% Inferior Temporal Gyrus, temporooccipital part |
| 2535 | 13.7 | -46 | -74 | -14 | 59% Lateral Occipital Cortex, inferior division, 17% Occipital Fusiform Gyrus |
| 260 | 7.23 | 10 | 28 | 36 | 39% Paracingulate Gyrus, 13% Cingulate Gyrus, anterior division |
| 248 | 6.05 | 34 | 20 | 6 | 39% Insular Cortex, 24% Frontal Operculum Cortex |
| 154 | 6.27 | -36 | 20 | 6 | 65% Frontal Operculum Cortex, 10% Insular Cortex |
| 47 | 5.73 | -22 | -64 | 48 | 60% Lateral Occipital Cortex, superior division, 5% Superior Parietal Lobule |
| 18 | 5.51 | -30 | -54 | 56 | 44% Superior Parietal Lobule, 7% Lateral Occipital Cortex, superior division |
| 10 | 5.7 | 6 | -30 | -2 | 65% Brain-Stem |
| 9 | 5.34 | 48 | 6 | 30 | 47% Precentral Gyrus, 8% Inferior Frontal Gyrus, pars opercularis |
| 8 | 5.35 | 38 | -2 | 58 | 37% Precentral Gyrus, 29% Middle Frontal Gyrus |
| 8 | 5.65 | -4 | -30 | -4 | 73% Brain-Stem |
| 8 | 5.67 | -46 | -12 | -4 | 52% Planum Polare, 12% Heschl's Gyrus (includes H1 and H2) |
| 6 | 5.26 | 30 | 48 | 26 | 86% Frontal Pole |
| 5 | 5.43 | 22 | -60 | 58 | 2% Lateral Occipital Cortex, superior division, 8% Superior Parietal Lobule |
| 3 | 5.18 | 38 | -6 | 50 | 37% Precentral Gyrus, 10% Middle Frontal Gyrus |
| 3 | 5.47 | -40 | 16 | -8 | 56% Insular Cortex, 9% Frontal Orbital Cortex |
| 3 | 5.8 | -40 | -52 | -32 | 96% Left Crus I |
| 2 | 5.13 | 18 | -68 | 56 | 48% Lateral Occipital Cortex, superior division |
| 2 | 5.07 | -26 | -60 | -12 | 42% Temporal Occipital Fusiform Cortex, 16% Occipital Fusiform Gyrus, 9% Lingual Gyrus |
| 2 | 5.29 | 30 | 44 | 40 | 77% Frontal Pole |
| 2 | 11.2 | 26 | -40 | -12 | 42% Lingual Gyrus, 24% Temporal Occipital Fusiform Cortex, 15% Parahippocampal Gyrus, posterior division, 11% Temporal Fusiform Cortex, posterior division |
| 1 | 5.1 | 30 | -60 | 62 | 51% Lateral Occipital Cortex, superior division, 13% Superior Parietal Lobule |
| 1 | 5.1 | -2 | -34 | -22 | 99% Brain-Stem |
| 1 | 5.28 | -2 | -42 | -14 | 6% Brain-Stem, 75% Left I-IV |
| 1 | 5.03 | 40 | -2 | 48 | 31% Precentral Gyrus, 24% Middle Frontal Gyrus |
| 1 | 5.03 | 40 | -2 | -14 | 46% Insular Cortex, 11% Planum Polare |
| 1 | 5.15 | 42 | -38 | 46 | 39% Supramarginal Gyrus, posterior division, 18% Superior Parietal Lobule, 7% Postcentral Gyrus |
| 1 | 5.07 | -18 | -78 | 42 | 57% Lateral Occipital Cortex, superior division, 9% Precuneus Cortex |
| 1 | 5.03 | -42 | 2 | 34 | 38% Precentral Gyrus, 22% Middle Frontal Gyrus |
| 1 | 5.92 | -48 | -8 | -4 | 43% Planum Polare, 18% Heschl's Gyrus (includes H1 and H2) |
| 1 | 5.11 | -4 | 32 | 26 | 47% Paracingulate Gyrus, 44% Cingulate Gyrus, anterior division |
| 1 | 10.1 | 18 | -28 | 0 | 88% Right Thalamus |
| 1 | 6.22 | 40 | 22 | 24 | 26% Middle Frontal Gyrus, 10% Inferior Frontal Gyrus, pars opercularis, 5% Inferior Frontal Gyrus, pars triangularis |
| 1 | 5.34 | 2 | 36 | 20 | 66% Cingulate Gyrus, anterior division, 16% Paracingulate Gyrus |
| 1 | 5.05 | 26 | 48 | 18 | 62% Frontal Pole |
| 1 | 5.11 | 46 | 16 | 12 | 29% Inferior Frontal Gyrus, pars opercularis |
| 1 | 5.01 | -48 | -24 | 10 | 59% Heschl's Gyrus (includes H1 and H2), 7% Planum Temporale, 7% Central Opercular Cortex |
| 1 | 9.19 | 42 | -2 | 0 | 75% Insular Cortex |
| 1 | 11.3 | -40 | -6 | 34 | 14% Precentral Gyrus |
| **Negative main effect of task** | | | | | |
| 333 | 6.18 | -48 | -62 | 24 | 40% Lateral Occipital Cortex, superior division, 34% Angular Gyrus |
| 9 | 5.21 | 12 | -50 | 38 | 42% Precuneus Cortex, 19% Cingulate Gyrus, posterior division |
| 8 | 5.53 | -24 | -52 | 22 | 2% Precuneus Cortex |
| 4 | 6.56 | -10 | -34 | 8 | 57% Left Thalamus |
| 3 | 5.49 | 48 | -74 | 46 | 1% Lateral Occipital Cortex, superior division |
| 2 | 5.55 | 34 | -52 | 4 | 2% Lingual Gyrus, 1% Precuneus Cortex |
| 1 | 13 | -14 | -40 | 36 | 14% Cingulate Gyrus, posterior division |
| 1 | 6.38 | 18 | -42 | 30 | 1% Cingulate Gyrus, posterior division |
| 1 | 9.05 | 34 | -52 | 24 | 4% Angular Gyrus |
| 1 | 6.78 | 40 | -52 | 22 | 18% Angular Gyrus |
| 1 | 7.07 | 38 | -48 | 8 | 1% Angular Gyrus, 1% Supramarginal Gyrus, posterior division |
| 1 | 7.41 | -68 | -52 | 2 | 22% Middle Temporal Gyrus, temporooccipital part |
| 1 | 5.48 | -4 | 48 | -14 | 83% Frontal Medial Cortex, 5% Paracingulate Gyrus |
| 1 | 5.03 | 22 | -80 | -36 | 95% Right Crus II |
| **Negative task*temperature interaction** | | | | | |
| 5 | 6.39 | 46 | 44 | 22 | 80% Frontal Pole |
| 1 | 5.27 | 42 | 20 | 2 | 63% Frontal Operculum Cortex, 8% Frontal Orbital Cortex, 5% Insular Cortex |
| 1 | 5.23 | 50 | 40 | 22 | 50% Frontal Pole, 13% Middle Frontal Gyrus |
| 1 | 5.09 | 56 | 26 | 32 | 19% Middle Frontal Gyrus |
| 1 | 5.52 | 52 | 34 | 32 | 12% Middle Frontal Gyrus, 5% Frontal Pole |
